# In-hospital free fatty acids levels predict the severity of myocardial ischemia of acute coronary syndrome

**DOI:** 10.1186/s12872-016-0199-1

**Published:** 2016-02-01

**Authors:** Pei Ma, Lu Han, Zhihua Lv, Wei Chen, Hanning Hu, Jiancheng Tu, Xin Zhou, Song-Mei Liu

**Affiliations:** Center for Gene Diagnosis, Zhongnan Hospital of Wuhan University, Donghu Road 169#, Wuhan, 430071 Hubei PR China

**Keywords:** Acute coronary syndrome, Free fatty acids, Gensini score

## Abstract

**Background:**

We aimed to assess whether the levels of FFAs (free fatty acids) in ACS (acute coronary syndrome) patients depend on the extent of myocardial ischemia during the subacute phase of ACS attack.

**Methods:**

A total of 892 consecutive CAD (coronary artery disease) subjects undergoing coronary angiography were enrolled. The FFAs contents were measured based on enzymatic assay. The relationship between FFAs and Gensini score and ACS susceptibility was assessed.

**Results:**

In the overall population, the upper FFAs quartile was accompanied with higher ischemia parameters and increased occurrence of ACS and STEMI (ST-segment elevation myocardial infarction) (*P* < 0.05). The FFAs concentrations were approximately 1.5-fold higher in ACS than in stable CAD patients, roughly 1.3-fold higher in STEMI than non-STEMI ACS patients and probably 1.3-fold higher in non-STEMI ACS than in stable CAD patients. After adjusted for traditional cardiovascular risk factors, the FFAs level remained a risk factor for a higher Gensini score with more than 40 (*P* < 0.001) and prevalent ACS (*P* < 0.001). After adjusted for traditional risk factors, FFAs levels after natural logarithm transformation were associated with hs-CRP and WBC counts in ACS patients. A multiplicative interaction was found between hs-CRP, WBC counts and FFAs in incident ACS and higher Gensini score (*P* < 0.001).

**Conclusions:**

Higher in-hospital levels of FFAs persist and may reflect the severity of ischemia and necrosis during the subacute phase of ACS attack.

**Electronic supplementary material:**

The online version of this article (doi:10.1186/s12872-016-0199-1) contains supplementary material, which is available to authorized users.

## Background

Plasma free fatty acids (FFAs) levels are elevated within 12 h after the onset of acute myocardial infarction (AMI) [1], moreover, elevated FFAs are associated with worse outcomes including increased risk of arrhythmia [2], sudden cardiac death and total mortality [3]. Although most FFAs are bound to albumin, a small fraction is unbound FFA (FFAu); FFAu increases in the ischemic, under-perfused myocardium rapidly within 30 min, which is considered to be more sensitive in physiologic changes than bound FFAs.

Inflammation plays a critical role throughout the atherosclerotic stage in various vessels, ranging from fatty streak formation to cardiovascular events [4, 5]. Among the potential inflammation biomarkers, C-reactive protein, the classical acute-phase protein, functions as a highly sensitive systemic marker of inflammation and tissue damage and can be measured by high-sensitivity assay (hs-CRP). Numerous epidemiological studies have demonstrated that hs-CRP had a clear prognostic value for predicting major cardiovascular events and mortality [6, 7].

Increased FFAu is an early predictor of ischemia in ACS (acute coronary syndrome) [8]. Our previous study found that plasma FFA compositions were dysregulated in CAD (coronary artery disease) patients, partly influenced by age and gene polymorphisms [9, 10]. Therefore the objective of our study was to explore the association between total FFAs and the severity of myocardial ischemia of ACS during subacute phase, and further investigated whether this was influenced by hs-CRP.

## Methods

### Subjects

The study was approved by the medical ethical committee of Zhongnan Hospital of Wuhan University. All participants were Han population from China, who gave their informed, written consent. From 2012 to 2014, we consecutively recruited patients who underwent selective coronary angiography for suspected coronary atherosclerosis and who were admitted within 24 h from chest pain at cardiovascular department. The diagnosis of CAD was defined as the presence of coronary lesions ≥50 % in at least one major artery segment assessed by two independent cardiologists who had no information of the patients’ clinical characteristics and biochemical results. We excluded all patients with additional complications including viral hepatitis, tumor, autoimmune disease, serious liver or renal dysfunction, myocardiopathy or valvular heart disease and patients received with heparin treatment.

Serial blood samples were taken after admission for routine laboratory parameter tests, including liver function, kidney function, lipids and lipoproteins and myocardial enzymes. Estimated glomerular filtration rate (eGFR) was calculated using the Modification of Diet in Renal Disease (MDRD) study equation: eGFR = 186 × ((serumcreatinine)^(‐ 1.154)) × ((age)^(‐0.203)) × [0.742 if female]. AMI was defined by typical clinical symptoms, electrocardiograph changes and positive serum biomarkers [11, 12]. Unstable angina (UA) was established based on the criteria of Brawmwald using clinical symptoms, non-ST segment elevation and negative serum biomarkers [13].

### Assay of FFAs content

Serum samples were collected, separated and stored at −80 °C within 24 h of admission. Samples were incubated for 15 min at room temperature and then a single determination of FFAs content was tested by enzymatic assay (Clinimate NEFA, Tokyo, Japan).

### Angiographic analysis

Catheterization and multiple views were imaged and maximal stenosis in each coronary artery segment was evaluated by a cardiologist according to the segmental classification system of the Coronary Artery Surgery Study. The extent of angiographically documented CAD was quantified in the left anterior descending artery, the left circumflex artery, and the right coronary artery, the criteria were as follows: normal coronary arteries (smooth, with either no stenosis or stenosis of <10 % of the luminal diameter), mild coronary arteries (stenosis of 10 % to 50 % of the luminal diameter in ≥ 1 coronary artery), or 1, 2 or 3-vessel disease, defined as stenosis of more than 50 % of the luminal diameter.

### Gensini score

To assess coronary artery disease burden based on coronary angiogram, the Gensini score was calculated according to Gensini scoring system [14]. The Gensini score equaled the sum of all segment scores (where each segment score equaled the segment weighting factor multiplied by the severity score). Severity scores assigned to the specific percentages of luminal diameter reduction of the coronary artery segment were 32 for 100 %, 16 for 99 %, 8 for 90 %, 4 for 75 %, 2 for 50 %, and 1 for 5 %.

### Statistical analysis

Variables were expressed as the mean ± SD, median (25th–75th percentile) or proportions and compared using Mann–Whitney *U*-test, Kruskal-Wallis H-test or the χ^2^ test in univariable analysis. Binary logistic regression analysis was used to identify correlated risk factors. All analyses were performed using PASW Statistics, version 13.0 (SPSS, Chicago, IL, USA).

## Results

### Clinical characteristic

The study population consisted of 404 SCAD (stable coronary artery disease) patients, including 294 chronic stable angina patients (male 56.1 %, aged 68 ± 11),110 old myocardial infarction patients (male 76.4 %, aged 66 ± 12) and 488 ACS patients, including 108 UA patients (male 56.6 %, aged 68 ± 11), 191 NSTEMI (non-ST segment elevation myocardial infarction) patients (male 76.2 %, aged 67 ± 13) and 189 STEMI (ST segment elevation myocardial infarction) patients (male 82.4 %, aged 62 ± 13).

The baseline demographic, clinical characteristics and laboratory parameters according to the quartiles of the FFAs levels (Q1 0.12–0.37, *n* = 223; Q2 0.38–0.54, *n* = 218; Q3 0.55–0.78, *n* = 228; Q4 0.79–4.57, *n* = 223) for the study population were summarized in Table 1. The distribution of FFAs quartiles were slightly unbalanced with traditional cardiovascular risk factors such as age, smoking, fasting blood glucose, hs-CRP and the atherogenic lipid parameters including TC, LDL-C, TG (*P* < 0.05). In addition, the necrosis and ischemia parameters dramatically increased according to FFAs quartiles such as cTnT, CK-MB, maximal stenosis and Gensini score (*P* < 0.05).

**Table 1:** Clinical characteristic of study population

	Q1 (*n* = 223)	Q2 (*n* = 218)	Q3 (*n* = 228)	Q4 (*n* = 223)	*P*
FFAs (mmol/l)	0.27 (0.21, 0.32)	0.46 (0.42, 0.49)	0.65 (0.59, 0.72)	1.01 (0.88, 1.28)	-
Demographics					
Age (y)	63 ± 13	60 ± 10.4	60 ± 10.4	62 ± 12	0.021
Male (%)	65.5	67.9	70.6	66.8	NS
Risk factors					
SBP (mmHg)	141 ± 24	133 ± 16	134 ± 21	124 ± 20	NS
DBP (mmHg)	82 ± 11	79 ± 11	82 ± 15	75 ± 11	NS
HR (bpm)	72 ± 7	74 ± 15	75 ± 8	74 ± 14	0.01
Smoking (%)	33.8	51.9	51.3	49.7	0.005
Hypertension (%)	67.7	69.3	65.4	57.4	0.043
Diabetes mellitus (%)	26.9	30.3	31.3	29.6	NS
Hyperlipidemia (%)	58.7	62.4	70.2	66.4	0.048
Laboratory parameter					
TC (mmol/l)	4.04 ± 0.86	4.28 ± 1.04	4.78 ± 1.56	4.3 ± 0.88	<0.001
HDL-C (mmol/l)	1 ± 0.2	1.05 ± 0.2	0.99 ± 0.21	1 ± 0.2	NS
LDL-C (mmol/l)	2.5 ± 0.92	2.73 ± 0.83	2.93 ± 0.8	2.75 ± 0.7	<0.001
Lp (a) (mg/l)	131 (67, 246)	78 (37, 312)	125 (61, 202)	136 (56, 209)	NS
TG (mmol/l)	1.63 ± 0.92	1.96 ± 1.37	2.35 ± 1.45	2.01 ± 1.6	<0.001
FBG (mmol/l)	5.96 ± 1.7	6.3 ± 2.6	7 ± 2.7	6.5 ± 3.3	<0.001
WBC (×10^9^)	7.5 ± 3.7	6.9 ± 2.2	8.1 ± 3.1	9.24 ± 3.2	<0.001
cTnT(ng/ml)	0.02 (0.003, 0.49)	0.07 (0.007, 2.84)	0.28 (0.01, 6.65)	1.72 (0.05, 15.1)	<0.001
hs-CRP (mg/l)	2.64 (0.58, 6.2)	2.42 (1.43, 3.83)	3.4 (1.16, 9.18)	2.43 (0.94, 11.7)	<0.001
CK-MB (U/l)	13 (8.5, 18.5)	13 (9, 26)	18 (11, 49)	31 (12, 102.8)	<0.001
eGFR (ml/min/1.73 m^2^)	93.4 ± 28.9	95.3 ± 20.2	98.2 ± 19.8	96.8 ± 30.8	NS
Angiographic analysis					
Number of vessel	2.2 ± 0.6	2.13 ± 0.9	2.24 ± 0.74	1.97 ± 0.86	NS
Maximal stenosis (%)	78.3 ± 16.8	81.2 ± 20	83.3 ± 18	84.6 ± 19.1	<0.001
Gensini score	22.9 ± 18	29.9 ± 28	37.9 ± 30	43.7 ± 41	0.001
Final diagnosis					
ACS n (%)	72 (8.1)	103 (11.5)	135 (15.1)	176 (19.7)	<0.001
STEMI n(%)	11 (1.2)	31 (3.5)	60 (6.7)	92 (10.3)	<0.001
Immediate therapy					
β-Blocker n (%)	43 (19.2)	43 (19.8)	39 (17.1)	36 (16.3)	NS
Aspirin n (%)	215 (96.3)	209 (95.7)	207 (91)	211 (94.5)	NS
Stent implantation n (%)	60 (27.1)	62 (28.2)	68 (29.7)	70 (31.4)	NS

Data were expressed as the mean ± SD, median (25th-75th percentile) or %.

*Q* = quartile, *SBP* systolic blood pressure, *DBP* diastolic blood pressure, *HR* heart rate

### Association of FFAs with severity of ischemia and necrosis

**Fig. 1:**
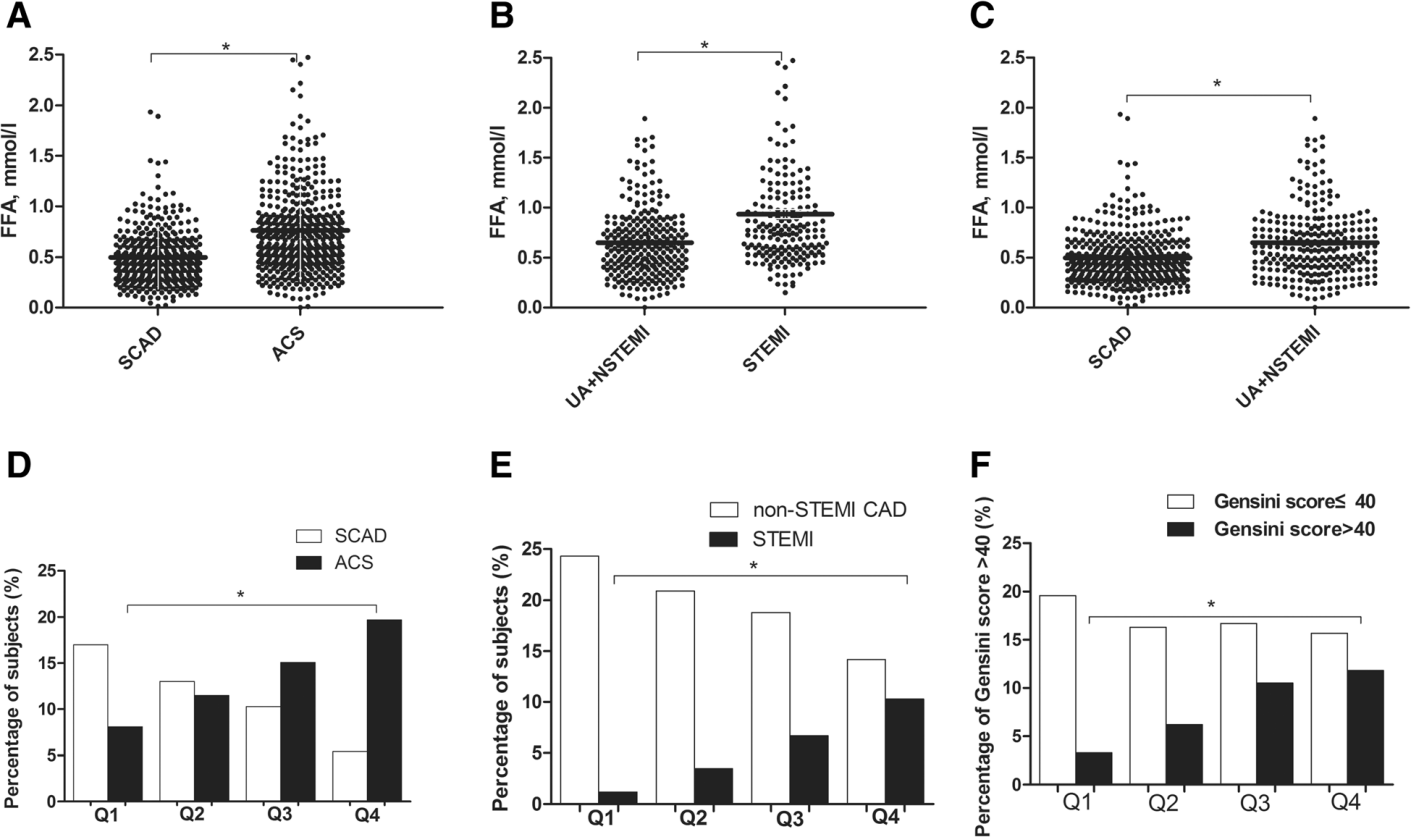
Distributions of FFAs among CAD patients (**a**, **b**, **c**) and prevalence of ACS, STEMI and Gensini score more than 40 based on the FFAs quartiles (**d**, **e**, **f**). Asterisks (*) represent a *P*-value <0.05. Q = quartile.

The FFAs levels in ACS patients were higher than in SCAD patients ((0.68 mmol/l (0.45, 0.911) vs. 0.44 mmol/l (0.3, 0.63), *P* < 0.001, respectively)) (Fig. 1a), which remained a significant correlation after adjustment for age and gender (*P* < 0.001). Among patients with ACS, the higher FFAs levels remained significant between STEMI and UA + NSTEMI patients ((0.77 mmol/l (0.55, 1.1) vs. 0.58 mmol/l (0.4, 0.83), *P* < 0.001, respectively)) (Fig. 1b), which was significant after adjustment for age and gender (*P* < 0.001). Meanwhile, the FFAs levels in UA + NSTEMI patients were higher than in SCAD patients ((0.58 mmol/l (0.4, 0.83) vs. 0.44 mmol/l (0.3, 0.63), *P* < 0.001, respectively)) (Fig. 1c), and the difference still remained significant after adjustment for age and gender (*P* < 0.001), suggesting that FFAs content increased closely with the severity of coronary artery lesions in CAD.

Meanwhile, the occurrence of ACS and STEMI increased according to the FFAs quartiles (*P* value for trend = 0.003, 0.001) (Fig. 1d, e). To explore the relationship of FFAs and the prevalent ACS and STEMI, a binary logistic regression analysis was performed. After adjusted for traditional cardiovascular risk factors including age, SBP, DBP, smoking, history of hypertension, diabetes mellitus and hyperlipidemia, the FFAs levels remained positively associated with prevalent ACS and STEMI ((for the occurrence of ACS, OR(95 % CI) = 9.956(5.21, 19.023), *P* < 0.001; for the occurrence of STEMI, OR(95 % CI) = 8.428(4.619, 15.376), *P* < 0.001)) (Tables 2 and 3).

**Table 2:** Association of FFAs levels and quartiles with prevalent ACS

	Unadjusted		Adjusted	
	OR (95 % CI)	*P*	OR (95 % CI)	*P*
FFAs	8.727 (5.331, 14.286)	<0.001	9.956 (5.21, 19.023)	<0.001
FFAs quartile				
Q1	1		1	-
Q2	1.833 (1.248, 2.693)	0.002	2.003 (1.201, 3.339)	0.008
Q3	1.811 (1.491, 2.199)	<0.001	2.081 (1.592, 2.72)	<0.001
Q4	1.988 (1.724, 2.292)	<0.001	2.189 (1.794, 2.671)	<0.001

Binary logistic regression analyses were performed. The adjusting confounders included age, gender, SBP, DBP, smoking, history of hypertension, diabetes mellitus and hyperlipidemia.

*Q* = quartile

**Table 3:** Association of FFAs levels and quartiles with prevalent STEMI

	Unadjusted		Adjusted	
	OR (95 % CI)	*P*	OR (95 % CI)	*P*
FFAs	6.634 (4.269, 10.31)	<0.001	8.428 (4.619, 15.376)	<0.001
FFAs quartile				
Q1	1		1	-
Q2	3.569 (1.709, 7.453)	<0.001	5.301 (1.702, 16.509)	0.004
Q3	2.736 (1.927, 3.885)	<0.001	3.53 (2.02, 6.169)	<0.001
Q4	2.419 (1.923, 3.042)	<0.001	3.083 (2.143, 4.436)	<0.001

Binary logistic regression analyses were performed. The adjusting confounders included age, gender, SBP, DBP, smoking, history of hypertension, diabetes mellitus and hyperlipidemia.

*Q* = quartile

To further explore whether the FFAs levels could reflect the severity of ischemia and necrosis focus in ACS patients, we performed Spearman’s correlation analysis. It showed that FFA level was positively correlated with Gensini score and maximal stenosis (r = 0.224, *P* < 0.001 and r = 0.224, *P* < 0.001) and remained significant after adjustment for age and gender (*P* = 0.001 and *P* = 0.003). The occurrence of a higher Gensini score with more than 40 increased according to the FFAs quartiles (*P* value for trend < 0.001) (Fig. 1f), after adjusted for traditional cardiovascular risk factors including age, SBP, DBP, smoking, history of hypertension, diabetes mellitus and hyperlipidemia, the FFAs levels remained a risk factor for a higher Gensini score with more than 40 ((OR(95 % CI) = 3.741 (1.826, 7.664), *P* < 0.001)) (Table 4). Correlations of FFAs content with the infarct size in ACS patients, estimated by CK-MB and cTnT were performed, showing that FFA levels had positively weak correlations with infarct size (r = 0.09, *P* < 0.001 and r = 0.022, *P* < 0.001).

**Table 4:** Association of FFAs levels and quartiles with Gensini score more than 40

	Unadjusted		Adjusted	
	OR (95 % CI)	*P*	OR (95 % CI)	*P*
FFAs	2.379 (1.393, 4.062)	<0.001	3.74 (1.826, 7.664)	<0.001
FFAs quartile				
Q1	1		1	-
Q2	2.538 (1.094, 5.889)	0.03	1.934 (0.688, 5.435)	NS
Q3	2.013 (1.358, 2.984)	<0.001	1.68 (1.029, 2.743)	0.038
Q4	1.704 (1.308, 2.22)	<0.001	1.605 (1.157, 2.223)	0.005

Binary logistic regression analyses were performed. The adjusting confounders included age, gender, SBP, DBP, smoking, history of hypertension, diabetes mellitus and hyperlipidemia.

*Q* = quartile

### Interaction between WBC, hs-CRP and FFAs

We constructed the multivariate linear regression analysis to elucidate the association of FFAs with inflammation parameters, after adjusting for traditional cardiovascular risk factors including age, SBP, DBP, smoking, history of hypertension, diabetes mellitus and hyperlipidemia, which showed after natural logarithm transformation that FFAs were associated with hs-CRP (β = 0.003, *p* = 0.048) and WBC (β = 0.042, *p* = 0.008), respectively.

**Fig. 2:**
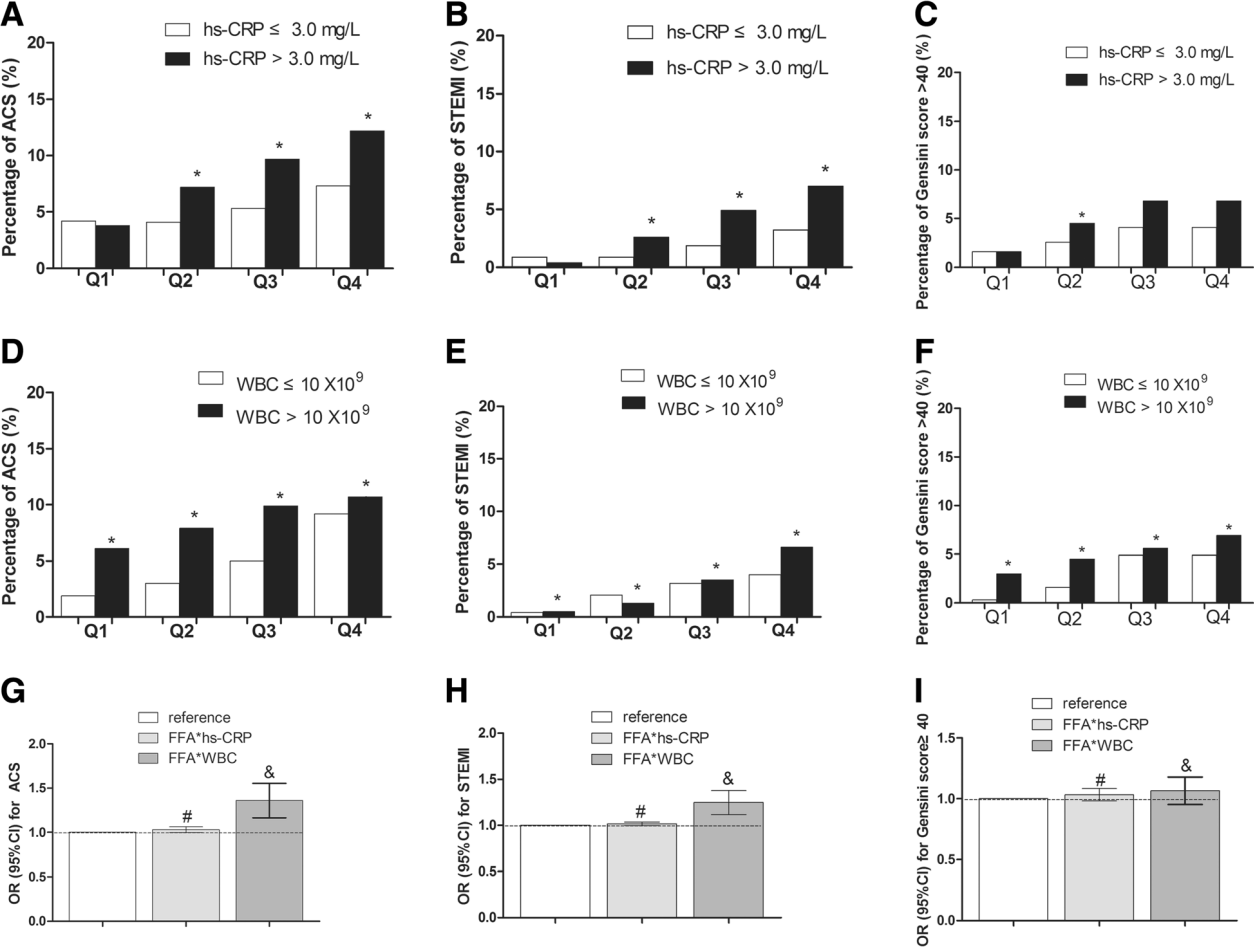
Interaction between WBC counts, hs-CRP and FFAs levels in ACS (a, d, g), STEMI (b, e, h), Gensini Score (c, f, i). Asterisks (*) showed significant difference in high/low hs-CRP and WBC levels by χ^2^ analysis. Pound key(#) represented significant difference in the comparison of interaction to non-interaction between FFAs and hs-CRP. Joint mark (&) showed significant difference in the comparison of interaction to non-interaction between FFAs and WBC counts. Q = quartile.

The whole study population were divided into four groups according to the FFAs quartiles and each group was sectionalized into 2 subgroups according to their high/low levels of WBC (≤10×10^9^, >10×10^9^) or hs-CRP (≤3.0 mg/l, >3.0 mg/l) respectively [15]. As shown in Fig. 2a, among all FFAs quartile groups, patients with hs-CRP more than 3.0 mg/l were more susceptible to ACS, compared to patients with hs-CRP less than 3.0 mg/l (*P* <0.05); the trend for ACS occurrence in both high and low hs-CRP groups was significantly elevated with the distribution of higher FFA level (*P* value for trend < 0.05) (Additional file 1: Figure S1A). The occurrence of STEMI was elevated with higher hs-CRP, except in the lowest FFAs quartile Q1 group (*P* < 0.05) (Fig. 2b); a similar trend was observed in STEMI susceptibility and FFAs level in both high and low hs-CRP groups (*P* value for trend < 0.05) (Additional file 1: Figure S1B). Meanwhile, higher hs-CRP level was associated with a higher Gensini score in the FFAs quartile Q2 group (*P* < 0.05) (Fig. 2c); the percentage of Gensini score for more than 40 dramatically increased according to FFAs quartiles in both high and lowe hs-CRP groups (*P* value for trend <0.001) (Additional file 1: Figure S1C). The similar trendsexist in WBC counts and ACS susceptibility (Fig. 2d, Additional file 1: Figure S1D), WBC counts and STEMI susceptibility (Fig. 2e, Additional file 1: Figure S1E), WBC counts and higher Gensini score (Fig. 2f, Additional file 1: Figure S1 F), respectively.

The trend of ACS occurrence, STEMI attack and higher Gensini score with higher FFAs in high hs-CRP and WBC levels versus low hs-CRP and WBC levels were suggestive of an interaction between hs-CRP, WBC counts and FFAs in relation to the progress of myocardial ischemia. After performed by logistic regression analysis, we found a multiplicative interaction between hs-CRP, WBC and FFAs in ACS and STEMI susceptibility and a higher Gensini score ((in relation to ACS susceptibility, FFAs*hs-CRP, OR(95 % CI) = 1.029(1.016, 1.043), *P* < 0.001, FFAs*WBC, OR(95 % CI) = 1.357(1.281, 1.438), *P* < 0.001; in relation to STEMI susceptibility, FFAs*hs-CRP, OR(95 % CI) = 1.016(1.008, 1.024), FFAs*WBC, OR(95 % CI) = 1.246(1.194, 1.3), *P* < 0.001; in relation to a higher Gensini score, FFAs*hs-CRP, OR(95 % CI) = 1.033(1.013, 1.054), *P* = 0.001, FFAs*WBC, OR(95 % CI) = 1.065(1.02, 1.112), *P* = 0.004)) (Fig. 2g, h, i), which suggests that FFAs influence the progress of ischemia involved by inflammation processes.

## Discussion

The major findings of our study confirmed that FFAs level might serve as a predictor of the severity of myocardial ischemia during the subacute onset of ACS attack. First and foremost, FFAs levels were higher in the ACS patients than the SCAD population, especially in STEMI patients. Second, the FFAs increased with the severity of necrosis and ischemia, such as cTnT and Gensini score. Moreover, we observed an association between WBC counts, hs-CRP and FFAs levels in incident ACS and higher Gensini score, which implied a possible interaction between FFAs and inflammation processes influenced the severity of ischemia.

According to the previous studies, elevated FFAs levels in AMI are associated with increased lipolytic activity, owing to an immediate increase of catecholamine with the activated sympathetic nervous system [16]. Although increasing evidence has shown that an elevation of FFAs level occurs after the onset of AMI and that higher FFAs are associated with a greater incidence of major cardiovascular events [17], no study has resolved the principal mystery whether FFAs directly trigger serious cardiovascular disease or only predict cardiometabolic dysfunction [18]. There are several mechanisms illustrating high FFAs concentration may be toxic in acute ischemic myocardium, such as mitochondrial uncoupling, activation of lipids in mitochondria, inhibition of β-oxidation, inhibition of the Na^+^-K^+^-ATPase pump leading to high intracellular sodium and calcium, or reduction of GLU-4 causing reduced insulin-stimulated glucose transport [19]. Therefore, it is a top priority to monitor and reduce concentrations of FFAs in the post phase of ACS [20].

Moreover, we found hs-CRP, WBC and FFAs had a multiple effect in relation to ACS susceptibility and severe myocardial ischemia (Fig. 2), which implied a possible mechanism relating FFAs together with inflammatory factors influenced the progress of ischemia. Inflammatory cytokines such as IL-6 and TNF-α were added to isolated adipocytes, resulting in increased lipolysis and FFAs levels in several in-vivo and in-vitro studies [21, 22], also, elevated circulating FFA levels led to endothelial dysfunction in-vivo via activation of PKC-mediated inflammatory pathways and excess generation of oxidants [23, 24], which would partially explain the clinically demonstrated proarrhythmogenic potential of FFAs. Further investigations in the underlying mechanisms for the joint effect of elevated FFA and inflammatory factors on myocardial ischemia are apparently warranted.

Interestingly, preexisting hypertension had been diagnosed in SCAD, ACS and STEMI patients with the prevalence of 70.5 %, 60.2 % and 48.1 %, respectively and that decreased ratio of hypertension was associated with higher occurrence of ACS and STEMI (Additional file 2). The role of hypertension history in the prevalence of ACS was difficult to explain. A large observational study showed that patients with preexisting hypertension had a more favorable in-hospital outcome [25]. Despite with a higher risk, hypertensive patients was paradoxically associated with a more favorable in-hospital diagnosis. Underlied the mystery, awareness and treatment of hypertension with potentially cardio-protective antihypertensive drugs might function as a ‘protective’ effect [25].

## Conclusions

FFAs levels may be indicative of the severity of myocardial ischemia at subacute ACS onset and the combined effect of FFAs and activated inflammation might play a role in myocardial ischemia of ACS.

## Additional files

Additional file 1: Figure S1. The occurrence of ACS, STEMI and percentage of Gensini score more than 40 according to high/low WBC and hs-CRP levels. Asterisks (*) showed significant difference among the FFAs quartiles by χ^2^ analysis. Q=quartile. (TIF 97015 kb) Additional file 2: Table S1. The results of χ^2^ analysis for occurrence of hypertension in study population (DOCX 13 kb)

## Abbreviations

ACS: acute coronary syndrome
AMI: acute myocardial infarction
CAD: coronary artery disease
DBP: diastolic blood pressure
FBG: fasting blood glucose
FFAs: free fatty acids
FFAu: unbound FFA
HDL-C: high density lipoprotein cholesterol
HR: heart rate
hs-CRP: high-sensitivity C-reactive protein
LDL-C: low density lipoprotein cholesterol
Lp(a): lipoprotein(a)
Q: quartile
SBP: systolic blood pressure
SCAD: stable coronary artery disease
STEMI: ST-segment elevation myocardial infarction
TC: total cholesterol
TG: triglyceride
UA: unstable angina
WBC: white blood cell
