# Supplementary material for: In-hospital free fatty acids levels predict the severity of myocardial ischemia of acute coronary syndrome
Source: BMC Cardiovasc Disord. 2016 Feb 1;16:29. doi: 10.1186/s12872-016-0199-1 (PMC4736147; doi:10.1186/s12872-016-0199-1)
Supplement: Additional file 2: Table S1. — The results of χ2 analysis for occurrence of hypertension in study population (DOCX 13 kb) [file 12872_2016_199_MOESM2_ESM.docx]

Table S1 The results of χ^2^ analysis for occurrence of hypertension in study population

| Groups | n (%) | OR (95% CI) | P |
| --- | --- | --- | --- |
| SCAD | 285 (70.5) | 1 | - |
| ACS | 294 (60.2) | 0.633 (0.478,0.838) | 0.001 |
| STEMI | 91 (48.1) | 0.388 (0.271,0.554) | <0.001 |

The occurrence of hypertension in SCAD was took as reference and other groups were compared to the reference by χ^2^ analysis.
